# Dr. Bell’s Select Medical Library

**Published:** 1844-08

**Authors:** 


DR. BELL'S SELECT MEDICAL LIBRARY.

We derive greater pleasure from none of our exchanges than from Dr. Bell's Bulletin and Select Medical Library. The Bulletin for July came accompanied by a volume of upwards of 500 pages, comprising the whole of Dr. Robert Lee's Lectures on the Theory and Practice of midwifery, delivered in St. George's Hospital, London. These lectures, which were first printed in the London Medical Gazette, have been revised by the author, and from his edition the work before us has been strictly copied without note or comment. We have not had time to read it carefully, but a cursory examination has satisfied us that it is the production of a highly cultivated and practical mind, and has been elaborated with a degree of judgment and care which must render the lectures a valuable record of professional learning and experience. The profession has a guaranty, in the excellent taste, and discriminating judgment of the editor of the Select Medical Library, that nothing will come forth under his auspices of a trashy, superficial, or spurious character. Y.

